# Adopting machine learning to automatically identify candidate patients for corneal refractive surgery

**DOI:** 10.1038/s41746-019-0135-8

**Published:** 2019-06-20

**Authors:** Tae Keun Yoo, Ik Hee Ryu, Geunyoung Lee, Youngnam Kim, Jin Kuk Kim, In Sik Lee, Jung Sub Kim, Tyler Hyungtaek Rim

**Affiliations:** 1 B&VIIt Eye Center, Seoul, South Korea; 2 Institute of Vision Research, Department of Ophthalmology, Yonsei University College of Medicine, Seoul, South Korea; 3 MediWhale, Seoul, South Korea; 4 Singapore Eye Research Institute, Singapore National Eye Centre, Duke-NUS Medical School, Singapore, Singapore; 5 Department of Ophthalmology, Yonsei University College of Medicine, Graduate School, Seoul, Korea

**Keywords:** Eye manifestations, Machine learning

## Abstract

Recently, it has become more important to screen candidates that undergo corneal refractive surgery to prevent complications. Until now, there is still no definitive screening method to confront the possibility of a misdiagnosis. We evaluate the possibilities of machine learning as a clinical decision support to determine the suitability to corneal refractive surgery. A machine learning architecture was built with the aim of identifying candidates combining the large multi-instrument data from patients and clinical decisions of highly experienced experts. Five heterogeneous algorithms were used to predict candidates for surgery. Subsequently, an ensemble classifier was developed to improve the performance. Training (10,561 subjects) and internal validation (2640 subjects) were conducted using subjects who had visited between 2016 and 2017. External validation (5279 subjects) was performed using subjects who had visited in 2018. The best model, i.e., the ensemble classifier, had a high prediction performance with the area under the receiver operating characteristic curves of 0.983 (95% CI, 0.977–0.987) and 0.972 (95% CI, 0.967–0.976) when tested in the internal and external validation set, respectively. The machine learning models were statistically superior to classic methods including the percentage of tissue ablated and the Randleman ectatic score. Our model was able to correctly reclassify a patient with postoperative ectasia as an ectasia-risk group. Machine learning algorithms using a wide range of preoperative information achieved a comparable performance to screen candidates for corneal refractive surgery. An automated machine learning analysis of preoperative data can provide a safe and reliable clinical decision for refractive surgery.

## Introduction

Recently, refractive surgery has produced excellent visual outcomes, and the number of refractive surgeries has grown.^1^ It has now become more important for the refractive surgeon to select candidates to undergo corneal refractive surgery in order to avoid complications.^2^ In order to minimize complications after surgery, the surgeon has to accurately examine the patient’s eyes to preoperatively identify cases with a likely poor outcome.

There are complicated relationships between optical parameters such as myopic level, pupil size, corneal radius, and ablation zone.^3^ When a clinician considers the optical parameters to improve visual quality, the preoperative corneal radius and sphericity were used in a calculation formula to obtain the postoperative corneal curvature.^4^ Age and refraction should also be considered as predictors of refractive stability after surgery.^5^ Because surgeons may find it hard to calculate all nonlinear relationships of optical variables to minimize the complication of each patient, the clinical decision was made based on the surgeon’s experience.

Ocular imaging technology has evolved in recent years to address candidacy issues in the corneal refractive surgery.^6^ A complete preoperative examination has to be performed, and the refractive surgeon should review all examination results before recommending a procedure. This can be a time-consuming process, and it is possible to overlook a sign of surgery contraindications. This is even more likely given the increasing workload for the refractive surgeon with the rise in population seeking refractive surgery. Up to now, there is still no definitive screening method to confront the possibility of a misdiagnosis.

Machine learning, which is an area of artificial intelligence research, has become popular in clinical medicine because of its ability to handle big data and to classify cases with high accuracy.^7^ Support vector machines (SVM), random forests (RF), artificial neural networks (ANN), AdaBoost, and least absolute shrinkage and selection operator (LASSO) are widely used approaches in machine learning.^8^ These techniques have been applied to many tasks in medicine and bioinformatics to select informative variables and predicting diagnoses more accurately.^9^ The current machine learning technique classified Pentacam-based corneal data with good performance for keratoconus diagnosis.^10^ A random forest model using Pentacam measurement data showed the good diagnostic accuracy to classify patients into stable cases and clinical ectasia after refractive surgery.^11^ Random forest was also used to combine the corneal biomechanical factors from Corvis ST (Oculus, Wetzlar, Germany).^12^ However, to our knowledge, the diagnostic value of the combination of all preoperative data has not been previously emphasized in the literature investigating patient selection using machine learning.

**Fig. 1:**
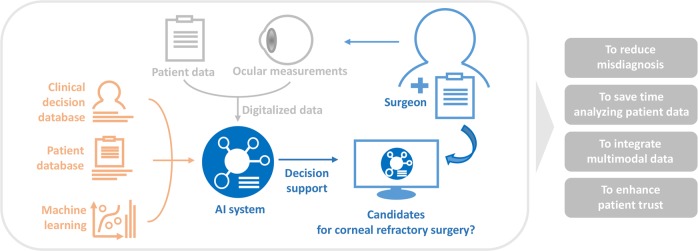
Schematic illustrating the purpose of this study

In our experience, surgeons or medical centers have slightly different criteria for corneal refractive surgery. Based on a clinical decision, refractive surgery can be performed in selected patients having a condition of relative contraindications, such as young adults, unstable refraction, large pupil size, dry eye, and diabetes. Moreover, surgeons should consider the nonlinear relationships of optical parameters to minimize the complication of each patient. Since all patient data and ocular measurements have been digitalized, the current technology can analyze the database to help refractive surgeons. For this study, we have built a machine learning architecture with the aim of identifying candidates for corneal refractive surgery to support clinical decision making (Fig. 1). The machine learning model was trained using clinical decisions of highly experienced experts. The employed architecture was based on large-sized preoperative clinical and ophthalmometric data and validated in a large Korean population indicated for refractive surgery.

## Results

### Characteristics of the study population

The background characteristics of the training and validation datasets are presented in Table 1. Among 18,480 screened subjects, a total of 1630 (8.8%) subjects were considered to have a contraindication to corneal refractive surgery. The comparison between the candidates and contraindication cases for surgery is presented in Supplementary Table 2.

**Table 1:** Characteristics of the subjects in this study for training and validation data

Variable	Training set (*N* = 10,561)	Internal validation set (*N* = 2640)	External validation set (*N* = 5279)	*P* value^a^
Age (years)	27.94 ± 6.12	27.89 ± 6.10	26.23 ± 6.51	<.001
Sex, female (%)	5609 (53.1)	1374 (52.0)	2879 (54.5)	.081
Spherical equivalent (Diopter)	−4.56 ± 2.24	−4.55 ± 2.20	−4.80 ± 2.28	<.001
CDVA (logMAR)	−0.015 ± 0.042	−0.016 ± 0.043	0.001 ± 0.041	<.001
IOP (mmHg)	15.20 ± 4.81	15.25 ± 5.47	15.16 ± 3.06	.008
Central corneal thickness (μm)	541.86 ± 31.54	541.82 ± 31.93	542.80 ± 33.38	.070
NIBUT (seconds)	6.87 ± 6.60	6.90 ± 6.67	6.83 ± 5.93	<.001
Corneal refractive surgery				
LASIK (%)	3630 (34.4)	914 (34.6)	1579 (29.9)	<.001
LASEK (%)	2891 (27.4)	729 (27.6)	1273 (24.1)	<.001
SMILE (%)	3036 (28.7)	746 (28.3)	2052 (38.8)	<.001
Contraindication cases for surgery (%)	1004 (9.5)	251 (9.5)	375 (7.1)	<.001

*CDVA* corrected distance visual acuity, *IOP* intraocular pressure, *LASEK* laser epithelial keratomileusis, *LASIK* laser in situ keratomileusis, *NIBUT* noninvasive break-up time, *SMILE* small incision lenticule extraction

^a^Comparison using the Kruskal−Wallis test and chi-square test

### Parameter and feature selection

**Fig. 2:**
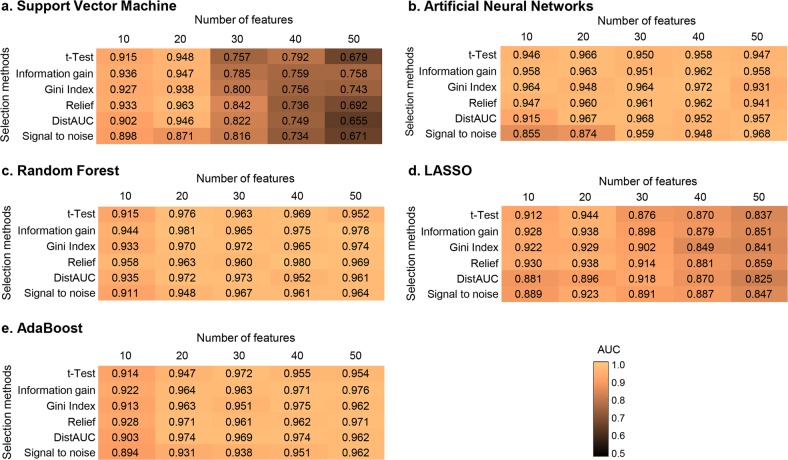
Heatmaps representing the predictive performance (AUC) of feature selection and machine learning methods to predict candidates for corneal refractive surgery. This figure shows the results from the tenfold cross-validation procedure. **a** Support vector machine. **b** Artificial neural networks. **c** Random forest. **d** Least absolute shrinkage and selection operator (LASSO). **e** AdaBoost

The optimal model of SVM was found using a Gaussian kernel function with a penalty parameter *C* of 1.0 and a scaling factor *γ* of 0.1. In RF, the optimal number of trees was 100, and the number of predictors for each node was 5. The optimal ANN was set with two hidden layers (100 and 2 nodes). In LASSO, the optimal sparseness parameter *λ* was 0.01. In AdaBoost, the decision tree was adopted as a weak estimator and the optimal number of estimator was 50. As shown in Fig. 2, the model with the highest performance was the RF model with 20 predictors selected by Information Gain (AUC = 0.981). As shown in Supplementary Fig. 1, Pentacam-based keratometry data (Pentacam_Cornea_Back_K1_right) was the highest ranked feature using Information Gain. Twelve of the top 20 ranked features were from the Pentacam corneal tomography.

### Algorithm performance

Table 2 shows the prediction performance via tenfold cross-validation of the machine learning and classic methods. After the feature selection, we obtained the AUCs of SVM, ANN, RF, AdaBoost, LASSO, and Ensemble of 0.963, 0.972, 0.981, 0.962, 0.938, and 0.983, respectively. The AUC of PTA (percentage of tissue ablated) and Randleman ectatic score were 0.827 and 0.897, respectively. The Delong test showed that RF outperformed SVM (*P* < .001), ANN (*P* = .004), AdaBoost (*P* < .001), LASSO (*P* < .001), PTA (*P* < .001), and Randleman ectatic score (*P* < .001). When the machine learning methods were combined using weighted majority voting, the performance was improved but it was not statistically significant (*P* = 0.579). Duncan’s multiple range test also showed that the machine learning methods with feature selection performed better than the classic methods.

**Table 2:** Classification performance of machine learning models to predict candidates for corneal refractive surgery using the tenfold cross-validation in the training set

	AUC (95% CI)	Accuracy (%) (95% CI)	Sensitivity (%) (95% CI)	Specificity (%) (95% CI)	*P* value^a^	Duncan subgroup^b^
Without feature selection						
SVM	0.612 (0.603–0.621)	55.2 (54.3–56.2)	54.7 (53.7–55.7)	60.8 (57.7–63.8)	<.001	G
ANN	0.824 (0.818–0.833)	75.1 (74.2–75.9)	75.3 (74.4–76.2)	72.9 (70.0–75.6)	<.001	F
RF	0.966 (0.963–0.970)	89.6 (88.9–90.1)	89.4 (88.8–90.0)	91.0 (89.1–92.7)	<.001	B, C
AdaBoost	0.962 (0.958–0.965)	89.0 (88.4–89.6)	89.0 (88.4–89.6)	89.2 (87.2–91.1)	<.001	B, C
LASSO	0.818 (0.811–0.825)	76.9 (76.1–77.7)	77.6 (76.7–78.4)	70.9 (68.0–73.7)	<.001	F
With feature selection						
SVM	0.963 (0.959–0.966)	90.1 (89.5–90.7)	90.2 (89.6–90.8)	89.6 (87.6–91.5)	<.001	C
ANN	0.972 (0.969–0.975)	91.8 (91.2–92.3)	91.9 (91.3–92.4)	90.7 (88.8–92.5)	.004	B
RF	0.981 (0.978–0.983)	92.7 (92.2–93.2)	92.6 (92.1–93.1)	93.6 (91.9–95.1)	Reference	A
AdaBoost	0.962 (0.958–0.965)	89.0 (88.4–89.6)	89.0 (88.4–89.6)	89.2 (87.2–91.1)	<.001	B, C
LASSO	0.938 (0.932–0.941)	87.3 (86.7–88.0)	87.5 (86.8–88.1)	86.0 (83.7–88.1)	<.001	D
Ensemble	0.983 (0.980–0.985)	94.3 (93.8–94.7)	94.5 (94.0–94.9)	92.5 (90.7–94.1)	.579	A
PTA	0.827 (0.820–0.835)	74.6 (73.7–75.4)	74.6 (73.7–75.5)	74.2 (71.4–76.9)	<.001	F
Randleman score	0.897 (0.892–0.903)	74.6 (73.7–75.4)	74.6 (73.7–75.5)	74.2 (71.4–76.9)	<.001	E

*ANN* artificial neural networks, *AUC* area under curve, *CI* confidence interval, *LASSO* least absolute shrinkage and selection operator, *PTA* percentage of tissue ablated, *RF* random forest, *SVM* support vector machine

^a^Comparison of receiver operating characteristics curves with the single best technique (random forest with feature selection) according to the Delong test

^b^The different letters (A, B, C, D, E, F, and G) indicate statistically different means according to Duncan’s multiple range test using the AUCs. The subgroup A (ensemble and random forest with feature selection) was significantly superior to other subgroups. The machine learning techniques with feature selection (A, B, C, and D) were significantly superior to the classic methods (E and F)

**Fig. 3:**
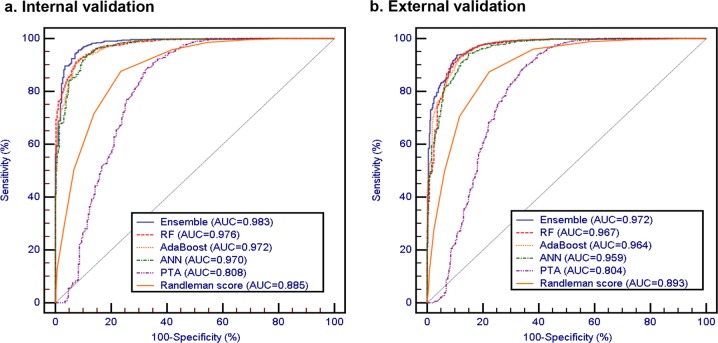
The ROC curves for the machine learning algorithms and classic screening methods. **a** The ROC curves of the internal validation set. **b** The ROC curves of the external validation set. The machine learning classifiers include random forest (RF), AdaBoost, artificial neural networks (ANN), and ensemble classifier. The classic methods include percentage of tissue ablated (PTA) and Randleman ectatic score

Figure 3 shows the ROC curves of machine learning models in predicting candidate subjects for corneal refractive surgery in the internal and external validation datasets. Supplementary Table 3 presents the detailed performance of the prediction models in the internal and external validation. This result was consistent with the cross-validation. The RF model was the single best discriminator among all techniques investigated but the differences were not statistically significant in comparison with other machine learning models. By combining all techniques, the ensemble method improved the performance in the internal (with the AUC of 0.983, 95% CI 0.977−0.987 and accuracy of 94.1%, 95% CI 93.2−95.0%) and external (with the AUC of 0.972, 95% CI 0.967−0.976 and accuracy of 93.4%, 95% CI 92.7−94.1%) validation, but the differences were again not statistically significant. The attached video shows the machine learning model as it appears during analysis (online Supplementary Video 1).

**Fig. 4:**
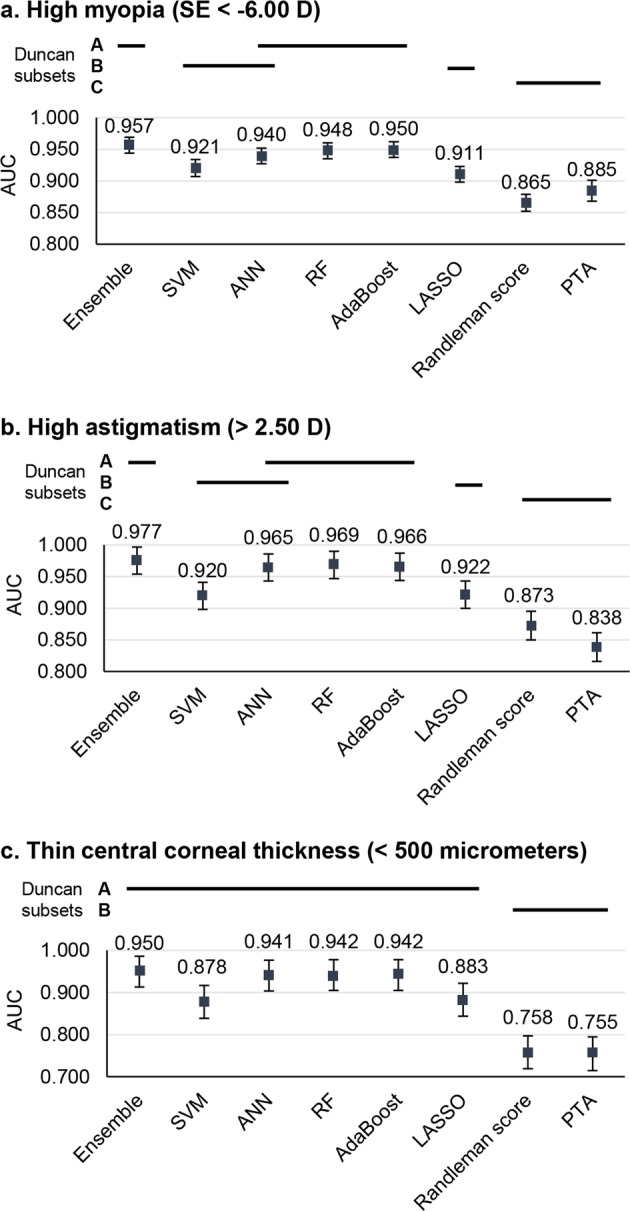
The classification performance of high-risk subgroups according to the tenfold cross-validation results. The performance was measured based on the average of the AUCs. The error bars represent the 95% confidence intervals. **a** Performances in the high myopia group. **b** Performances in the high astigmatism group. **c** Performances in the thin corneal thickness group. Error bars indicate the standard deviation of the mean

The prediction performance in the high-risk subgroups is presented in Fig. 4. The results show that ensemble, ANN, RF, and AdaBoost performed robustly in all subgroup having high myopia, high astigmatism, and thin central corneal thickness. Duncan’s multiple range test shows that the machine learning models with feature selection were superior to classic methods in all high-risk subgroups.

**Fig. 5:**
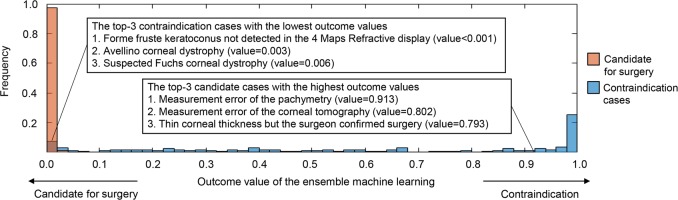
Outcome value histograms of the ensemble machine learning technique in the tenfold cross-validation. The misclassified samples with an opposite outcome value are shown

Figure 5 presents the outcome value histograms of the ensemble machine learning technique in the tenfold cross-validation. The misclassified samples with opposite outcome values showed that cases with forme fruste keratoconus not detected in 4 Maps Refractive Display, Avellino corneal dystrophy, and suspected Fuchs’ corneal dystrophy contributed to incorrect classifications in the contraindication group. By contrast, measurement errors in the pachymetry and corneal tomography (reexamination data were confirmed by surgeon) as well as operations confirmed by the surgeon despite a thin central corneal thickness contributed to incorrect classifications of candidates for surgery.

**Fig. 6:**
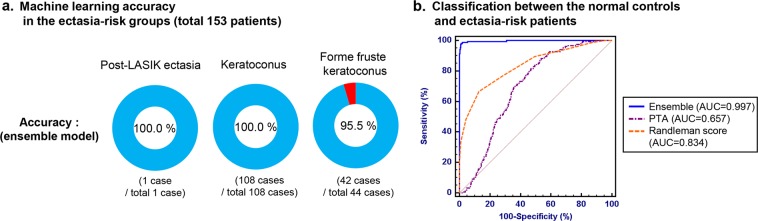
Machine learning technique performance in the ectasia-risk groups, including post-LASIK ectasia, keratoconus, and forme fruste keratoconus patients. **a** Accuracy in each ectasia-risk group. **b** ROC curves for classification between the normal control (no postoperative ectasia, *N* = 9556) and total ectasia-risk group (*N* = 153)

During the study period, post-LASIK ectasia was developed in one patient among the development dataset with follow-up data. The machine learning was able to reclassify this patient correctly as an ectasia-risk group (Fig. 6). One patient with post-LASIK ectasia, 108 patients diagnosed with keratoconus, and 44 patients with forme fruste keratoconus were included in the ectasia-risk group. The normal control group consisted of the subjects with normal preoperative measurements except one patient with post-LASIK ectasia. The ensemble machine learning model classified the ectasia-risk patients with an AUC of 0.996.

## Discussion

The current study aimed to automatically screen candidates for corneal refractive surgery using machine learning. The individual analysis of visual characteristics of the patient is most important for refractive surgery. Since we collected large cohort data, the machine learning algorithms could find the pattern of clinical and optical information. The machine learning architecture predicted candidates with an accuracy of 93.4% and an AUC of 0.972 in external validation. It combined the large multi-instrument data from patients and clinical decisions of highly experienced experts. These results show that the machine learning model performed as well as experts with a consistent performance in the high-risk subgroups with high myopia, high astigmatism, and thin central corneal thickness.

Our study suggests that machine learning-assistance during the preoperative evaluation will result in fewer missed contraindication cases. Nowadays, the gold standard to identify candidate cases for refractive surgery is subject to each clinician, although numerous articles described indications and contraindications to refractive surgery.^13^ Previous machine learning studies have focused patient data from one equipment.^11^ Our architecture using large multi-instrument data is closer to clinicians and easier to understand the outcome of the machine learning model. Computer-aided decision making could potentially eliminate the possibility of inter-clinician variability in selecting candidates for surgery. However, the machine learning performance may vary due to the reference decisions of the expert ophthalmologists in the training dataset. Despite this limitation, the artificial intelligence described in the present study could still be used to support the clinical decisions because the models predict with an objective and statistical background as a safeguard.

Although the machine learning models in this study were trained to imitate the expert ophthalmologists, the preoperative identification of forme fruste keratoconus is key for screening candidates for surgery. Recently, identifying subjects with ectatic predispositions has been crucial in corneal refractive surgery. The classic methodology for screening uses corneal tomography and central corneal thickness.^14^ Randleman et al. proposed an ectasia-risk score system for LASIK candidates based on corneal tomography, central corneal thickness, level of correction, residual stromal bed, and age.^15^ However, there is an important limitation related to the subjectivity for corneal tomography classification included in the Randleman ectatic score.^16^ Recently, optical coherence tomography was applied for a higher accuracy in the diagnosis,^17^ and biomechanical properties measurement of the corneal tissue using the Corvis ST has been adopted,^18^ but there is still no definitive screening parameter using these methods. Another potential solution to reduce both the interobserver variability and the likelihood of a misdiagnosis without employing newly developed devices is to apply a computerized analysis using machine learning techniques.

Machine learning approaches have been developed by incorporating preoperative information from different domains including visual acuity, refractive, and corneal tomography. In this study, machine learning models using decision trees including RF and AdaBoost performed better than other complex methods. RF was found to be a robust and accurate machine learning classifier in the previous literature.^19^ As a nonparametric statistical method, RF can deal with nonlinearity, interactions between predictors, and heterogeneity of predictors.^20^ AdaBoost is also a very popular technique and has been applied with great success in many pattern classification problems.^21^ Although AdaBoost is limited by combining weak learners, it can greedily select important features and can take a complicated problem by building sparse classification rules.^8^ We observed that the ensemble classifier using a weighted majority vote successfully boosted the performance in our study. The improvement was derived from the fusion of heterogeneous classifiers that might complement each other in an ensemble method.^22^

Differing from our comprehensive approach, several studies have emphasized the optimization of specific postoperative outcomes to improve visual quality.^23,24^ Previous researches have tried to minimize differences between the real ablation and the predicted one using a quadratic term in the formulae.^25^ The recent study demonstrated that a surgeon should consider the nonlinear relationships between optical variables using the Q-optimized algorithm.^26^ We believe that the machine learning technique will help specific problems in optimized refractive surgery, since it is optimal to build a nonlinear pattern model.

Several limitations should be noted. First, our study did not compare our proposed methods to the Belin−Ambrósio Enhanced Ectasia Display (BAD-D) index, which has been widely used to screen for keratoconus. The BAD-D was reported as a very accurate index in predicting ectasia risk.^27^ In our B&VIIT Eye Center, BAD-D was only calculated when a keratoconus was suspected. Since a previous study showed that the combination of BAD-D with other clinical measurements improves the accuracy,^28^ we expect that a machine learning model that includes BAD-D may boost the performance to predict candidates for refractive surgery. We also did not compare our methods to the Topographic and Biomechanical Index (TBI), which is based on random forest technique. The TBI measured using Corvis ST was excluded because it was applied since 2017 in the B&VIIT Eye Center. Second, the postoperative outcome data were not analyzed in this study. In fact, expert ophthalmologists are unable to forecast whether a complication will occur. We expect a longitudinal study design that includes the postoperative data to be a more powerful prediction tool to confidently differentiate candidates for corneal refractive surgery. Third, the specular endothelial cell count and the presence of corneal dystrophy were not included in our analysis. A definite diagnosis of corneal dystrophy needs slit-lamp examination and genetic evaluation. Unfortunately, these data were not standardized in our electronic health records, and they were, therefore, impossible to incorporate into our models. Our results demonstrate that Avellino and Fuchs’ corneal dystrophy definitely contributed to incorrect classifications. Fourth, this study was conducted in a single Asian country. Generally, the incidence of keratoconus is influenced by the genetic background.^29^ Therefore, it cannot be confirmed whether our proposed model can be applied to other ethnic groups or other eye clinics.

In conclusion, we have demonstrated that machine learning algorithms using a wide range of preoperative information yielded a performance comparable to that of screening for corneal refractive surgery. Our proposed machine learning model is expected to perform reliably, because it was trained by a large population. An automated analysis of preoperative data can provide a safe and reliable clinical decision for refractive surgery. In the future, this approach will facilitate standardized and automated selections of surgical choices.

## Methods

### Data source

This retrospective study protocol was approved by the Institutional Review Board of Korean National Institute for Bioethics Policy (KoNIBP, 2018-2734-001), which waived the requirement for informed consent. This study adhered to the tenets of the Declaration of Helsinki. This analysis included 18,480 healthy Korean subjects who intended to undergo refractive surgery at the B&VIIT Eye Center from January 2016 to June 2018. All patients underwent preoperatively measurements of best-corrected distance visual acuity and manifest refraction, slit-lamp examinations of the anterior segment, and dilated fundus examinations. Corneal tomography was measured using a Pentacam Scheimpflug device (Oculus Optikgeräte GmbH, Wetzlar, Germany). Pachymetry (NT-530P; Nidek Co., Ltd., Aichi, Japan) was used to evaluate the central corneal thickness. Pupil size and noninvasive tear break-up time (NIBUT) were also determined. Each subject was interviewed and asked to complete a split questionnaire survey about his or her occupation, anticipated surgery option, anticipated recovery period after surgery, and medical history. The detailed questionnaires are presented in Supplementary Table 1.

### Reference standard

All patients who had laser epithelial keratomileusis (LASEK), laser in situ keratomileusis (LASIK), or small incision lenticule extraction (SMILE) were considered as candidate subjects for corneal refractive surgery. General criteria for consideration for surgery, which may vary in several items from criteria used in other refractive practices, included the following parameter: age 18 years or older; myopia spherical equivalent > −10.0 diopters (D); hyperopia spherical equivalent < +4.50 D; central corneal thickness, measured with pachymetry, >500 μm for LASIK and >480 μm for LASEK and SMILE; residual corneal thickness > 380 mm after surgery, NIBUT > 5 seconds for LASIK; and absence of corneal abnormalities suggestive of keratoconus or other corneal ectatic diseases. These were not absolute criteria, and expert ophthalmologists could recommend corneal refractive surgery based on their clinical decision. A reference standard was assigned based on the clinical decision obtained from a full evaluation by nine experts. Basically, one surgeon was involved in the initial screening process for each patient. Two surgeons were involved in the assessment of complicated cases. Disagreement was resolved through discussion and data review. All experts were board-certified ophthalmologists with an average experience of 10 years in refractive surgery. An ophthalmologic examination was performed on all patients at postoperatively at 1 week and 1 month to screen postoperative ectasia.

### Machine learning techniques

**Fig. 7:**
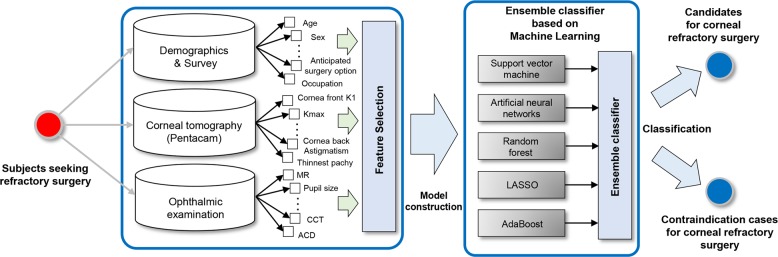
An architecture of our proposed machine learning system to predict candidates for corneal refractive surgery

A flow diagram of our proposed method is shown in Fig. 7. The machine learning models were designed to predict candidates for corneal refractive surgery. SVM is based on mapping data to a higher dimensional space through a kernel function and choosing the maximum-margin hyperplane that separates training data.^30^ RF is an ensemble learning classification method, which consists of a collection of decision trees and can deal in training with high-dimensional data faster than other methods with a very robust performance.^31^ ANN uses mathematical systems that mimic biological neural networks. We employed a multilayer perceptron neural network with back-propagation for nonlinear pattern classification.^9^ AdaBoost is a technique combining a set of weak learners to build a strong classifier.^32^ It always chooses the weak classifier with the lowest error, ignoring all others. LASSO is widely used as a sparse learning tool in bioinformatics.^33^ It leads to a sparse solution of coefficients corresponding to the most important predictors and has been known to show better performance for the prediction model selection and better identification of predictors than classical regression. Additionally, an ensemble classifier with a combination of all the above-mentioned machine learning techniques was built to improve the accuracy. We employed the weighted majority vote ensemble which is the most intuitive and widely used combiner.^34^

### Feature selection

In this study, the wide range of clinical and measurement data provides a highly redundant feature space. Supplementary Table 1 lists the 142 variables based on the demographics data, survey, corneal tomography, and other ophthalmic examinations. Eighty features from the corneal tomography on both eyes were automatically extracted from the 4 Maps Refractive Display using an in-house developed optical character recognition algorithm, which simply converted digits in a Pentacam image into text data. Therefore, we ranked features based on feature relevance and found the optimal subset for each machine learning technique. Filtering feature selection methods in this study were the *t* test,^35^ Gini index,^36^ Information Gain,^37^ Relief,^38^ DistAUC,^39^ and Signal to noise.^40^ For each feature ranking method, we varied the selection size and fitted machine learning classifiers on the selected feature subset using the training dataset. The performance of each classifier was determined by measuring the area under the receiver operating characteristic (ROC) curve (AUC). Keratoconus, which is the most important status for refractive surgery, present bilateral, but asymmetrically progressive thinning of the cornea. Therefore, measurements of both eyes should be included in the analysis.

### Model building and validation

A total of 13,201 subjects (71.4% of the enrolled subjects), who visited the Eye Center between January 2016 and December 2017, were used as the development dataset. The development dataset was separated randomly into training and internal validation sets. The training set, comprised of three fourths (10,561 subjects) of the entire development dataset, was used to construct the prediction models. The internal validation set, comprised of one fourth (2640 subjects) of the dataset, was used to assess the ability to predict eligible patients for corneal refractive surgery. In order to obtain an unbiased prediction, the performances of the prediction models were evaluated in data collected between January and June 2018. In this procedure, the external validation set comprised of a total 5279 subjects (28.6% of the enrolled subjects). In the training dataset, we designed a tenfold cross-validation, which is currently the preferred technique in data mining, not only to assess performance but also to optimize the prediction models. To obtain the optimal result, we adopted a grid search in which a range of parameter values was tested using the tenfold cross-validation strategy.

To validate the proposed models in high-risk groups, additional analyses were conducted in the high-risk subgroups, which included subjects with high myopia (spherical equivalent < −6.00 D), high astigmatism (cylinder diopter of refraction > 2.50 D), and thin central corneal thickness (<500 μm).^41^ The performance based on the high-risk subgroups were extracted from the tenfold cross-validation results. The developed model was also validated in differentiating the ectasia-risk group from normal controls with follow-up data. All patients with postoperatively diagnosed ectasia, preoperatively diagnosed keratoconus and forme fruste keratoconus were included in the ectasia-risk group.

In addition, the percentage of tissue ablated (PTA) and the Randleman ectatic score were calculated for all subjects in the study.^42,43^ PTA has been a simple and robust risk factor for ectasia after LASIK when corneal tomography is normal. The Randleman ectatic score provides a discrete risk scoring system with a comprehensive screening approach. Corneal tomography classification in the Randleman ectatic score was conducted subjectively by ophthalmologists according to the literature.^15^ For eyes with asymmetrical scoring, the worst-affected eye was considered. These classic screening methods were compared to our proposed prediction models.

### Statistical analysis

MATLAB 2017a (Mathworks, Natick, MA, USA) and R version 3.5.1 (The Comprehensive R Archive Network; http://cran.r-project.org) were adopted to perform the algorithms. MedCalc 12.3 (MedCalc, Mariakerke, Belgium) was used to conduct analyses of the ROC curves. To generalize the superiority of a classifier, inferential statistics should be conducted. Therefore, the comparison between AUCs used the nonparametric empirical method of Delong, which provides confidence interval and standard error of the difference between two AUCs. When a cross-validation was performed, Duncan’s multiple range test, which is a widely used test for multiple mean comparisons, was adopted to obtain detailed information about the differences between classifiers.^44^ This test identified the subsets of adjacent means that are different within a given level of significance (*α* < .05).^45^

### Reporting summary

Further information on research design is available in the Nature Research Reporting Summary linked to this article.

## Supplementary information

reporting summary

Supplementary materials

Supplementary video

## Data Availability

The data are not easily redistributable to researchers other than those engaged in the Institutional Review Board-approved research collaborations with the B&VIIt Eye Center, South Korea. The datasets utilized during this study are not publicly available due to reasonable privacy and security concerns.

## Supplementary information

**Supplementary information** accompanies the paper on the *npj Digital Medicine* website (10.1038/s41746-019-0135-8).

## Code availability

The underlying architecture is copyrighted by the B&VIIt Eye Center and will not be available to public. All code that was written to process and analyze the data can be available upon reasonable request from the corresponding author.
