# Supplementary material for: Adopting machine learning to automatically identify candidate patients for corneal refractive surgery
Source: NPJ Digit Med. 2019 Jun 20;2:59. doi: 10.1038/s41746-019-0135-8 (PMC6586803; doi:10.1038/s41746-019-0135-8)
Supplement: Supplementary file 2 — Supplementary materials [file 41746_2019_135_MOESM2_ESM.pdf]

Online-only supplements

Supplementary Table 1 Subjects' data used to build the machine learning models

Supplementary Table 2 Comparison between candidates and contraindication cases

Supplementary Table 3 Classification performance of machine learning models to predict candidates for corneal refractive surgery in the internal validation set

Supplementary Fig. 1 Twenty features were selected by information gain ranking to predict candidates for corneal refractive surgery. This graph shows the results from the 10-fold cross-validation procedure. CCT, central corneal thickness; CDVA, corrected distance visual acuity; K1, keratometry in the flattest meridian; K2, keratometry in the steepest meridian.

Supplementary Video This video demonstrates the final ensemble machine learning model as it appears during analysis.

Supplemental materials

Adopting Machine Learning to Automatically Identify Candidate Patients for Corneal Refractive Surgery

Tae Keun Yoo, MD, Ik Hee Ryu, MD, Geunyoung Lee, BS, Youngnam Kim, MS, Jin Kuk Kim, MD, PhD, In Sik Lee, MD, PhD, Jung Sub Kim, MD, Tyler Hyungtaek Rim, MD, MBA

Supplementary Table 1. Subjects' data used to build the machine learning models

Category	Total number	Features
Demographics & Survey	40	Age (continuous)<br>Sex (binary)<br>Before_Surgery_Glasses (binary)<br>Before_Surgery_Hard_Lens (binary)<br>Before_Surgery_Soft_Lens (binary)<br>Before_Surgery_None (binary)<br>Occupation_Sports (binary)<br>Occupation_Driver (binary)<br>Occupation_Computer_or_Smartphone (binary)<br>Anticipated_Surgery_LASIK (binary)<br>Anticipated_Surgery_LASEK (binary)<br>Anticipated_Surgery_SMILE (binary)<br>Anticipated_Surgery_ICL (binary)<br>Anticipated_Surgery_None (binary)<br>Anticipated_Recovery_One_Day (binary)<br>Anticipated_Recovery_Three_Days (binary)<br>Anticipated_Recovery_One_Week (binary)<br>Anticipated_Recovery_One_Month (binary)<br>Anticipated_Recovery_None (binary)<br>Plan_After_Surgery_Study_Abroad (binary)<br>Plan_After_Surgery_Employment (binary)<br>Plan_After_Surgery_Military (binary)<br>Plan_After_Surgery_Surgery (binary)<br>Plan_After_Surgery_None (binary)<br>Concern_Complication (binary)<br>Concern_Visual_Acuity (binary)<br>Concern_Management (binary)<br>Concern_Recovery (binary)<br>Concern_Money (binary)<br>Concern_None (binary)<br>Dry_Eye_Symptom_Severe (binary)<br>Dry_Eye_Symptom_Moderate (binary)<br>Dry_Eye_Symptom_Mild (binary)<br>Dry_Eye_Symptom_None (binary)<br>History_Metabolic_Disease (binary)<br>History_Glaucoma_Or_Retinal_Disorder (binary)<br>History_Keloid_Or_Atopic_Dermatitis (binary)<br>History_Recent_Delivery (binary)<br>History_Other (binary)<br>History_None (binary)

eTable 1. Subjects' data used to build the machine learning models (continued)

Category	Total number	Features
Corneal tomography - Pentacam (both eyes)	80	Pentacam_Pupil_Diameter (continuous)<br>Pentacam_Anterior_Chamber_Depth (continuous)<br>Pentacam_Angle (continuous)<br>Pentacam_Chamber_Volume (continuous)<br>Pentacam_Keratometric_Power_Deviation (continuous)<br>Pentacam_Corea_Volume (continuous)<br>Pentacam_K_Max_y (continuous)<br>Pentacam_K_max_x (continuous)<br>Pentacam_K_max_pachy (continuous)<br>Pentacam_Thinnest_Y (continuous)<br>Pentacam_Thinnest_X (continuous)<br>Pentacam_Thinnest_CCT (continuous)<br>Pentacam_Pachy_Apex_Y_Position (continuous)<br>Pentacam_Pachy_Apex_X_Position (continuous)<br>Pentacam_Pachy_Apex_CCT (continuous)<br>Pentacam_Pupil_Center_Y (continuous)<br>Pentacam_Pupil_Center_X (continuous)<br>Pentacam_Pupil_Center_CCT (continuous)<br>Pentacam_Corneal_Back_Rmin (continuous)<br>Pentacam_Corneal_Back_Rper (continuous)<br>Pentacam_Corneal_Back_ecc (continuous)<br>Pentacam_Corneal_Back_Astig (continuous)<br>Pentacam_Corneal_Back_Axis (continuous)<br>Pentacam_Corneal_Back_K_mean (continuous)<br>Pentacam_Corneal_Back_R_mean (continuous)<br>Pentacam_Corneal_Back_K2 (continuous)<br>Pentacam_Corneal_Back_R_Vertical (continuous)<br>Pentacam_Corneal_Back_K1 (continuous)<br>Pentacam_Corneal_Back_R_Horizontal (continuous)<br>Pentacam_Corneal_Front_Rmin (continuous)<br>Pentacam_Corneal_Front_Rper (continuous)<br>Pentacam_Corneal_Front_ecc (continuous)<br>Pentacam_Corneal_Front_Astig (continuous)<br>Pentacam_Corneal_Front_Axis (continuous)<br>Pentacam_Corneal_Front_K_mean (continuous)<br>Pentacam_Corneal_Front_R_mean (continuous)<br>Pentacam_Corneal_Front_K2 (continuous)<br>Pentacam_Corneal_Front_R_Vertical (continuous)<br>Pentacam_Corneal_Front_K1 (continuous)<br>Pentacam_Corneal_Front_R_Horizontal (continuous)
Ophthalmic examination (both eyes)	22	Spherical_Equivalent (continuous)<br>Spherical_Diopter (continuous)<br>Cylinder_Diopter (continuous)<br>Cylinder_Axis (continuous)<br>CDVA (logMAR) (continuous)<br>Pupil_Diameter (continuous)<br>IOP (continuous)<br>CCT (continuous)<br>Anterior_Chamber_Depth (continuous)<br>WTW (continuous)<br>NIBUT (continuous)
Total	142 features	

Supplementary Table 2. Comparison between candidates and contraindication cases

	Candidates for surgery	Contraindication cases	*P* Value[a]
**Training set**			
Number	9557	1004	
Age (years)	27.8 ± 5.9	33.8 ± 7.7	<.001
Sex, female (%)	5077 (53.1)	532 (53.0)	.935
Spherical equivalent (Diopter)	-4.53 ± 2.00	-7.82 ± 4.99	<.001
CDVA (logMAR)	-0.016 ± 0.036	0.017 ± 0.114	<.001
IOP (mmHg)	15.2 ± 2.59	15.2 ± 23.2	.897
Central corneal thickness (μm)	542.9 ± 30.58	501.3 ± 41.2	<.001
NIBUT (seconds)	6.93 ± 6.71	5.12 ± 3.60	<.001
**Internal validation set**			
Number	2389	251	
Age (years)	27.7 ± 6.1	33.6 ± 7.9	<.001
Sex, female (%)	1242 (52.0)	132 (52.6)	.856
Spherical equivalent (Diopter)	-4.53 ± 2.00	-7.82 ± 4.99	<.001
CDVA (logMAR)	-0.017 ± 0.037	0.015 ± 0.119	<.001
IOP (mmHg)	15.3 ± 3.6	15.2 ± 23.2	.923
Central corneal thickness (μm)	542.8 ± 30.97	501.7 ± 42.2	<.001
NIBUT (seconds)	6.91 ± 6.63	5.10 ± 3.44	<.001
**External validation set**			
Number	4904	375	
Age (years)	25.7 ± 6.4	33.8 ± 8.4	<.001
Sex, female (%)	2675 (54.6)	204 (54.4)	.956
Spherical equivalent (Diopter)	-4.57 ± 2.12	-7.81 ± 5.10	<.001
CDVA (logMAR)	-0.001 ± 0.036	0.019 ± 0.127	<.001
IOP (mmHg)	15.2 ± 3.1	15.1 ± 26.2	.917
Central corneal thickness (μm)	545.1 ± 32.8	509.8 ± 45.9	<.001
NIBUT (seconds)	6.94 ± 6.71	5.40 ± 3.60	<.001

Abbreviations: CDVA, corrected distance visual acuity; IOP, intraocular pressure; NIBUT, non-invasive break up time.[a] Comparison using the one-way ANOVA test and Chi-square test.

Supplementary Table 3. Classification performance of machine learning models to predict candidates for corneal refractive surgery in the internal validation set

	AUC (95% CI)	Accuracy (%) (95% CI)	Sensitivity (%) (95% CI)	Specificity (%) (95% CI)	*P* Value[a]
Internal validation					
SVM	0.963 (0.955-0.970)	90.6 (89.5-91.7)	90.7 (89.5-91.8)	90.0 (85.7-93.5)	.085
ANN	0.970 (0.962-0.976)	92.7 (91.7-93.7)	93.1 (92.0-94.1)	89.2 (84.7-92.8)	.357
RF	0.976 (0.969-0.981)	91.4 (90.3-92.4)	91.3 (90.3-92.4)	92.4 (88.4-95.4)	Reference
AdaBoost	0.972 (0.965-0.978)	92.1 (90.9-93.1)	92.1 (90.9-93.2)	91.6 (87.5-94.8)	.591
LASSO	0.930 (0.920-0.940)	84.7 (83.2-86.0)	84.5 (82.9-85.9)	86.5 (81.6-90.4)	<.001
Ensemble	0.983 (0.977-0.987)	94.1 (93.2-95.0)	94.3 (93.3-95.2)	92.8 (88.9-95.7)	.223
PTA	0.808 (0.792-0.822)	85.3 (83.9-86.6)	87.1 (85.7-88.4)	68.5 (62.4-74.2)	<.001
Randleman score	0.885 (0.872-0.897)	86.6 (85.2-87.8)	87.6 (86.2-88.9)	76.5 (70.7-81.6)	<.001
External validation					
SVM	0.958 (0.952-0.963)	91.5 (90.7-92.2)	91.7 (90.9-92.5)	88.0 (84.3-91.1)	.459
ANN	0.959 (0.953-0.964)	90.6 (89.7-91.3)	90.7 (89.9-91.5)	88.0 (89.9-91.5)	.503
RF	0.967 (0.962-0.972)	92.9 (92.2-93.6)	93.3 (92.5-93.9)	88.0 (84.3-91.1)	Reference
AdaBoost	0.964 (0.958-0.969)	90.6 (89.8-91.4)	90.6 (89.8-91.4)	90.4 (86.9-93.2)	.612
LASSO	0.913 (0.905-0.920)	85.5 (84.5-86.4)	85.7 (84.7-86.7)	82.7 (78.5-86.4)	<.001
Ensemble	0.972 (0.967-0.976)	93.4 (92.7-94.1)	93.7 (93.0-94.4)	89.3 (85.8-92.3)	.226
PTA	0.804 (0.958-0.969)	80.9 (79.9-82.0)	81.7 (80.6-82.8)	70.9 (66.1-75.5)	<.001
Randleman score	0.893 (0.884-0.901)	86.9 (85.9-87.8)	87.5 (86.6-88.5)	77.6 (73.0-81.7)	<.001

Abbreviations: ANN, artificial neural networks; AUC, area under curve; CI, confidence interval; LASSO, least absolute shrinkage and selection operator; PTA, percentage of tissue ablated; RF, random forest; SVM, support vector machine.

[a] Comparison of receiver operating characteristics curves with the best single technique (random forest with feature selection) according to the Delong test.

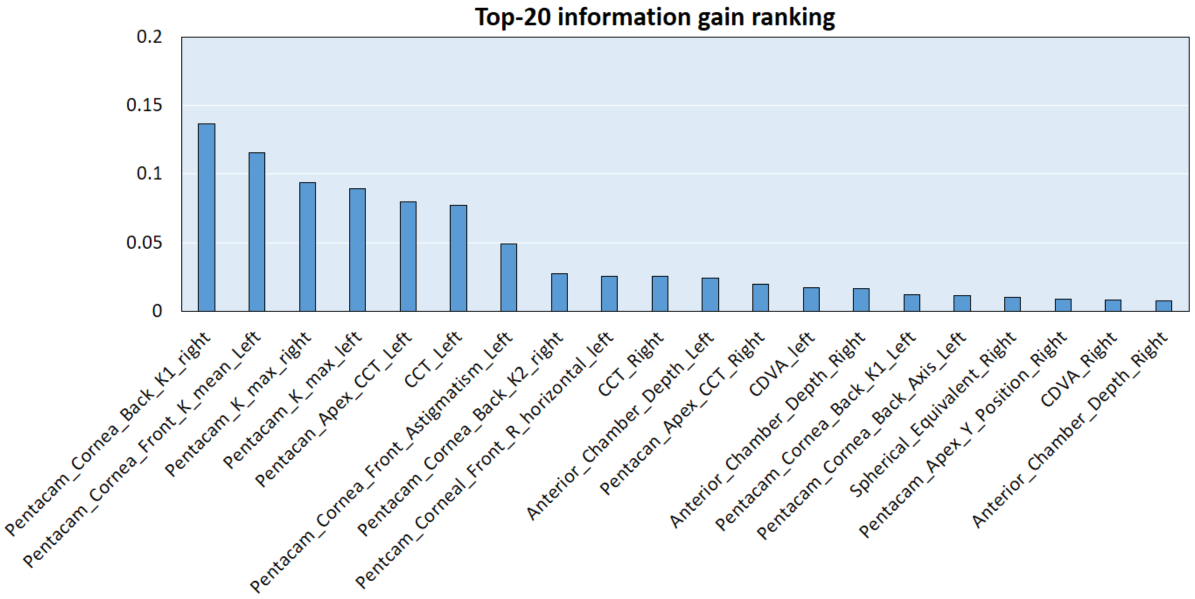

Supplementary Fig. 1. Twenty features were selected by information gain ranking to predict candidates for corneal refractive surgery.

This graph shows the results from the 10-fold cross-validation procedure. CCT, central corneal thickness; CDVA, corrected distance visual acuity; K1, keratometry in the flattest meridian; K2, keratometry in the steepest meridian
